# Role of CD8-positive cells in radioimmunotherapy utilizing ^177^Lu-mAbs in an immunocompetent rat colon carcinoma model

**DOI:** 10.1186/s13550-014-0079-6

**Published:** 2015-02-12

**Authors:** Erika Elgström, Sophie E Eriksson, Tomas G Ohlsson, Rune Nilsson, Jan Tennvall

**Affiliations:** Division of Oncology and Pathology, Department of Clinical Sciences, Lund University, Barngatan 2B, SE-221 85 Lund, Sweden; Department of Medical Radiation Physics, Lund University, Lund, Sweden; Department of Oncology, Skåne University Hospital, Lund, Sweden

**Keywords:** CD8 depletion, Cytotoxic T cells, Radioimmunotherapy, Immunocompetent animal model, Metastases

## Abstract

**Background:**

CD8-positive cells might play a crucial role in the therapeutic response to radiation, which has however not been investigated in radioimmunotherapy (RIT). The aim of this study was to evaluate whether cytotoxic T cells affect the response of established tumors and, above all, if they delay or prevent the development of distant metastases after RIT, using an immunocompetent syngeneic rat colon carcinoma model.

**Methods:**

The cytotoxic T cells were depleted in 15 rats by anti-CD8 before the injection of the radioimmunoconjugate (400 MBq/kg body weight ^177^Lu-BR96, which binds to the tumor-associated antigen Lewis Y). Fifteen other rats were treated with RIT only. Both groups were followed for 99 days. Blood samples were collected at least once weekly, and tumors were monitored twice weekly.

**Results:**

Twenty-nine of the 30 animals exhibited local complete response. The non-responder was treated with anti-CD8 and RIT but succumbed later due to metastases. Five animals in the group given anti-CD8 + RIT were sacrificed due to metastatic disease, and 4 additional animals were found to have metastases at autopsy. In the group given RIT, 4 animals developed metastatic disease, but no metastases were found in the remaining 11 animals at autopsy. Thus, at the end of the study, 6 animals in the anti-CD8 + RIT group were free from metastases, while 11 were free from metastases in the group receiving RIT.

CD3^+^CD4^−^CD8^+^ lymphocytes were consistently depleted by the anti-CD8 treatment. The myelosuppression was otherwise similar in the two groups. The initial depletion of CD8-positive cells in our syngeneic rat colon carcinoma model resulted in a higher frequency of animals developing metastases.

**Conclusions:**

Depletion of CD8-positive cells during RIT in an immunocompetent rat tumor model might influence the number of animals developing metastases, indicating that the immune system may be important in the long-term outcome of RIT.

## Background

The treatment of primary tumors is often successful, while metastatic disease still poses a considerable challenge. The vast majority of cancer-related deaths are the direct or indirect effect of distant metastases [1]. Novel modes of treatment are therefore needed to reduce or delay the development and growth of distant metastases.

Radioimmunotherapy (RIT) utilizes antibodies to direct radionuclides to specific antigens within the tumor. Using our immunocompetent syngeneic rat colon carcinoma model, we have previously shown that RIT consisting of the beta emitter ^177^Lu (400 MBq/kg body weight) conjugated to the monoclonal antibody BR96 resulted in complete response in 17 of 19 animals [2]. In the same study, cyclosporin A, a substance known to have its major effects on T cells [3-8], was given to prevent the development of immune response to the therapeutic mouse/human chimeric antibody. Treatment with cyclosporin A prolonged the time to complete response compared to animals treated with RIT only, but the fraction of animals exhibiting complete response was similar. Neither the time to metastatic disease nor the fraction of animals developing disseminated disease was affected by cyclosporin A. This observation indicates that T cells might be involved in the response process of RIT in our model.

Others have shown that the depletion of cytotoxic T cells by the administration of anti-CD8 antibodies reverses the anti-tumor effects of external-beam radiation therapy [9-12], which indicates that CD8-positive cells play a crucial role in the therapeutic response to radiation in these models. These findings motivated us to perform the present study to evaluate the effects of cytotoxic T cells on the response of established tumors and to establish whether they prevented the development of distant metastasis after RIT in our immunocompetent syngeneic rat colon carcinoma model. Half of the animals were given anti-CD8 on two occasions and all the animals were given RIT (400 MBq/kg body weight ^177^Lu-BR96). To the best of our knowledge, the role of CD8-positive cells during RIT has not previously been investigated.

## Methods

### Anti-CD8 antibodies

The OX-8 cell line (Health Protection Agency Culture Collections) producing a murine anti-CD8 monoclonal antibody was grown in CD Hybridoma Medium (Gibco, Life Technologies, Carlsbad, CA, USA) supplemented with 2 mM l-glutamine and 2.8 mg/L gentamicin (PAA Laboratories GmbH) for 10 to 12 days. The medium was centrifuged, and the supernatant was collected and stored at −20°C. The antibodies were purified utilizing HiTrap™ Protein A HP columns (GE Healthcare Life Sciences, Uppsala, Sweden), and the eluted antibodies were transferred to phosphate-buffered saline (PBS, Sigma-Aldrich, St. Louis, MO, USA) by repeated centrifugation using an Amicon-15 filter unit (molecular weight cutoff 30,000, Millipore, Billerica, MA, USA). The purity of the antibody solution was analyzed by bioanalyzer and a Protein 230 plus kit (Agilent Technologies, Santa Clara, CA, USA). The protein concentration was determined using the BCA (bicinchoninic acid) standard assay (Sigma-Aldrich, St. Louis, MO, USA). The purified antibody solution was stored at −20°C.

### The therapeutic monoclonal antibody

The chimeric (mouse/human) monoclonal IgG1 antibody BR96 (Seattle Genetics Inc., Bothell, WA, USA), which binds to the tumor-associated antigen Lewis Y, was used in this study. The Lewis Y antigen is expressed on the majority of human epithelial tumors, but normal human tissue also contains the BR96-binding antigen, primarily in the epithelial cells of the gastrointestinal tract [13]. The binding affinity between BR96 and the carcinoma cell line used in this study is strong (the dissociation constant being 4 nM) [14].

### Radioimmunoconjugation

Conjugation was performed according to Forrer *et al.* [15]. Briefly, BR96 was transferred to 0.2 M sodium carbonate buffer, pH 9.5, by repeated centrifugation using the Amicon-15 filter unit. The DOTA-chelate (S-2-(4-isothiocyanatobenzyl)-1,4,7,10-tetraazacyclododecane tetraacetic acid; 2 mg/mL H_2_O, Macrocyclics, Dallas, TX, USA) was added to the BR96 antibody (100 mg/mL) at a molar ratio of 3:1 (DOTA:BR96) and incubated for 1 hour at 37°C. The conjugate was purified by repeated centrifugation as described above and transferred to 0.25 M ammonium acetate buffer, pH 5.3. The final concentration was adjusted to 10 mg/mL BR96 by the addition of ammonium acetate buffer. All vials were pretreated with 1% HNO_3_ and all buffers were pretreated with Chelex-100 (Bio-Rad, Hercules, CA, USA) to remove metals.

MALDI-MS was used to determine the number of DOTA moieties per BR96 molecule, by desalting the sample to 18 MΩ · cm H_2_O using a centrifugation filter device, and dividing the increase in molecular mass by the molecular mass of the DOTA-chelate (688 u).

Both the ^177^LuCl_3_ solution (MDS Nordion, Ottawa, Canada) and the DOTA-BR96 conjugate in 0.25 M ammonium acetate buffer were preheated to 45°C for 10 min. The DOTA-BR96 solution was added to the vial containing the radionuclide and incubated at 45°C for 15 min. The reaction was quenched with an excess of DTPA (diethylene triamine pentaacetic acid) for 5 min. The radiolabeled immunoconjugate was diluted in 1% human serum albumin (HSA, Baxter, Deerfield, IL, USA) to prevent radiolysis from affecting the immunoreactivity. The radiochemical purity was determined by instant thin-layer chromatography (ITLC) using a 1 × 9 cm silica-gel-impregnated fiberglass sheet as the solid phase and 0.1 M EDTA as the mobile phase. To confirm the radiochemical purity and to detect signs of aggregation or fragmentation, separation was performed using size-exclusion chromatography and high-performance liquid chromatography (HPLC) (using a 7.8 × 300 mm molecular sieving column, Phenomenex SEC S3000 (Phenomenex, Torrance, CA, USA), eluted with 0.05 M sodium phosphate at 1.0 mL/min).

### Syngeneic animal model

BN7005-H1D2 is a cell line established from a 1,2-dimethylhydrazine-induced rat colon carcinoma in the Brown Norway (BN) rat. The cells were cultured in RPMI-1640 medium supplemented with 10% fetal calf serum, 1 mM sodium pyruvate, 10 mM HEPES buffer, and 14 mg/L gentamicin (all from PAA Laboratories GmbH) at 37°C, in a humidified environment containing 5% CO_2_. The cells were washed in PBS and detached by treatment with trypsin (both from PAA Laboratories GmbH). We have previously determined the radiosensitivity of this cell line, expressed as the fraction of survival after exposure to 2 Gy (S_2Gy_), to be 0.5 (^137^Cs radiation source, unpublished data). This is similar to the radiosensitivity of human colorectal carcinoma cell lines [16].

BN rats are immunocompetent and express the BR96 binding antigen in normal tissues, mainly in the epithelium of the gastrointestinal tract [17], similar to humans. The animals were inoculated with 3 × 10^5^ cells between the peritoneum and the abdominal wall under anesthesia (Isoflurane, Baxter). All experiments were conducted in compliance with European legislation on animal welfare and were approved by the Regional Animal Ethics Committee. The animals were housed under standard conditions and fed with standard pellets and fresh water *ad libitum*.

### Radioimmunotherapy and depletion of CD8-positive cells

The amount of administered anti-CD8 required for efficient depletion, suggested by Holmdahl *et al.* and Huang *et al.* [18,19], was confirmed by i.v. injection of 0.5 or 1.0 mg anti-CD8 (in 0.4 mL saline) in 6 tumor-free BN rats. The animals were followed for 25 days p.i., and blood samples were collected twice a week.

On day 0 (13 to 14 days after cell inoculation), 15 tumor-bearing BN rats in the RIT group, were given 400 MBq/kg ^177^Lu-BR96 (150 μg DOTA-BR96 in 0.4 mL saline with 1% HSA) by intravenous injection. In the anti-CD8 + RIT group, 15 tumor-bearing BN rats were given anti-CD8 on day −3 (0.5 mg in 0.4 mL saline) and day 9 (0.3 mg in 0.4 mL saline), and 400 MBq/kg body weight ^177^Lu-BR96 day 0. The tumors were measured using a caliper, and the tumor volumes were calculated as (tumor length × tumor width^2^ × 0.4) [20]. The median tumor volume of all animals on day 0 was 680 mm^3^ (interquartile range 460 mm^3^), and the median body weight on day 0 was 272 g (interquartile range 22 g). Body weight and tumor volume were measured twice a week. Blood samples were collected from all animals to monitor myelosuppression and for flow cytometric analyses of CD8-positive cells twice weekly from one week before injection and during the first 4 weeks after injection of the radioimmuno-conjugate, and then once weekly until the end of the study (day 99) or sacrifice. The study was ended 99 days p.i., as in our previous study lasting 180 days all metastatic disease was detectable within 100 days p.i. [21].

Animals were sacrificed and dissected if the tumor size approached 20 × 20 mm, if the decrease in body weight approached 20% of the normal weight progression, if the general health of the animal was affected during the study, or if metastatic disease was suspected. All remaining animals were sacrificed and dissected at the end of the study (day 99). All dissections were performed by the same person (EE) and the location of metastases was recorded.

### Blood analyses

Blood samples were used to evaluate the total number of white blood cells, as a measure of myelosuppression, and the CD3^+^CD4^−^CD8^+^ lymphocytes in order to monitor CD8-positive cells. Myelosuppression was determined by counting the white blood cells and platelets with a Vet CA530 Medonic Cell Analyzer (Boule Medical, Spanga, Sweden).

In order to label CD3/CD4/CD8a in blood, EDTA-treated blood samples were incubated with mouse anti-rat CD32 (BD Pharmingen) for 15 min to achieve FcR blockage and stained with FITC-conjugated anti-rat CD3, PE-conjugated anti-rat CD4, and Alexa Fluor 647-conjugated anti-rat CD8a (all from Biolegend, San Diego, CA, USA) for 30 min. Red blood cells were lysed with Erythrolyse Red Blood Cell Lysing Buffer (AbD Serotec, Oxford, UK), and fixed in 0.5% PFA (paraformaldehyde) in PBS/BSA (10 g BSA in 1 L PBS). Labeled cells were analyzed on the same day.

Samples were analyzed with a FACSCalibur flow cytometer (BD Bioscience, San Jose, CA, USA) using CellQuest Pro collection software (BD Bioscience). Corrections were made for spectral overlap. One hundred thousand events per sample were recorded. The collected data were analyzed with Flowing Software 2.5.0 (P. Terho, University of Turku, Finland). CD3^+^ lymphocytes were gated using forward scatter, side scatter, and FITC channels. CD4^−^CD8^+^ cells were counted in both the lymphocyte gate (in the forward scatter and side scatter plot) and CD3^+^ lymphocyte gate.

### Statistical analysis

The risk of developing metastases was calculated using a log-rank test (Cox-regression) with the STATA software (StataCorp LP, College Station, Texas, USA), while time to complete response of the local tumor (Mantel-Cox test) was calculated using Prism 5.02 (GraphPad Software Inc., La Jolla, CA, USA).

## Results

### Radioimmunoconjugate

The number of DOTA moieties per BR96 molecule was 2.6. After radiolabeling, the specific activity of the ^177^Lu-DOTA-BR96 conjugate was 675 MBq/mg. ITLC showed the radiochemical purity to be 95% and 4% aggregates or fragments were observed with HPLC.

### Local tumor response

**Figure 1:**
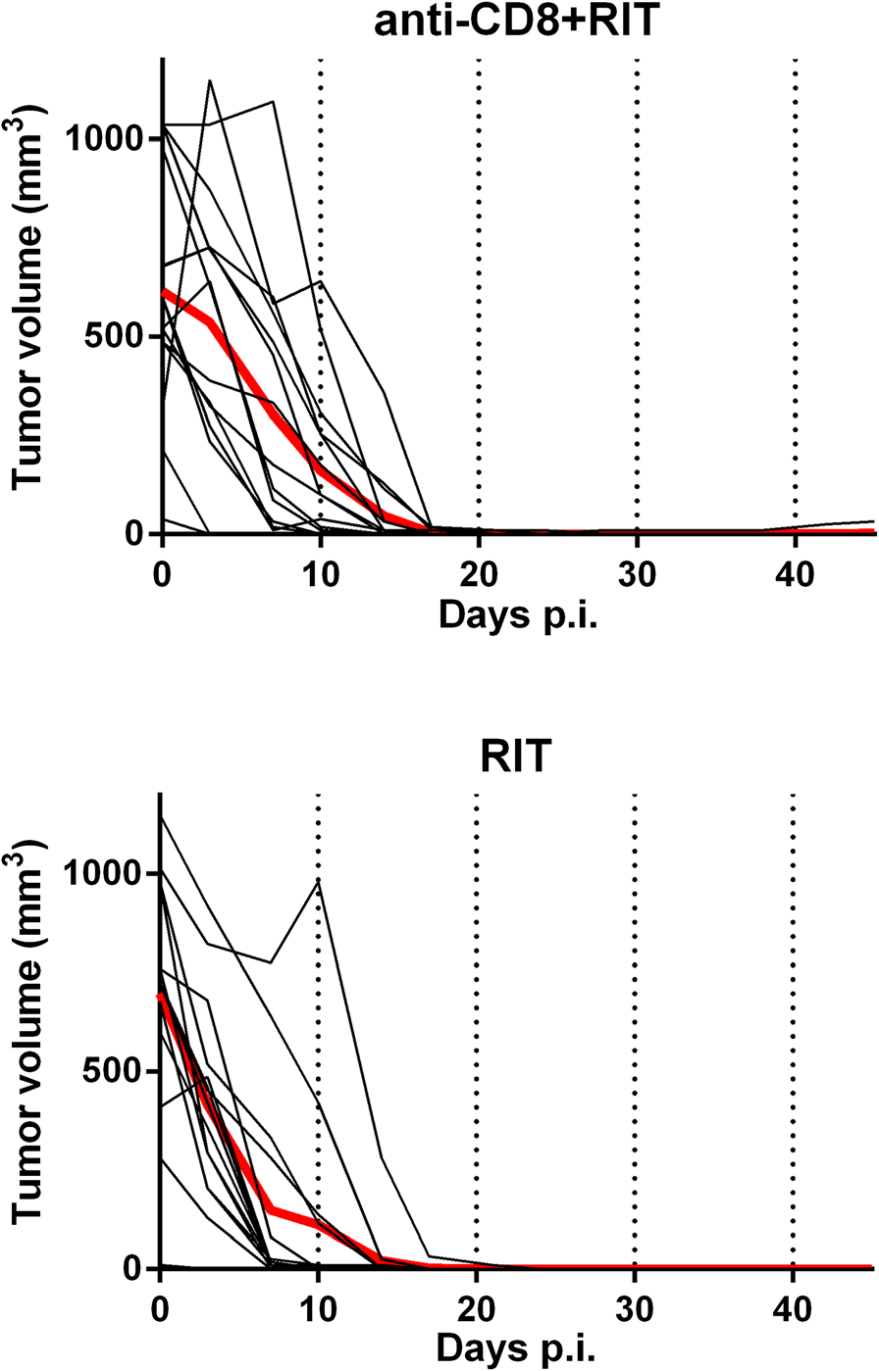
**Tumor volume growth after treatment.** Individual (*black*) and average (*red bold*) tumor volumes in animals treated with anti-CD8 + RIT (*above*) and in animals treated with RIT only (*below*).

All animals in the RIT group exhibited local complete response, defined as non-palpable tumor and no detectable tumor at dissection. In the anti-CD8 + RIT group, all animals but one showed local complete response, although this tumor initially responded well to the treatment (reduction in tumor volume of 99%). The animal not showing local complete response was sacrificed due to metastatic disease on day 45. There was no statistical difference between the groups regarding time to local complete response (Mantel-Cox test). Individual tumor volumes are presented in Figure 1.

### Disseminated disease

**Figure 2:**
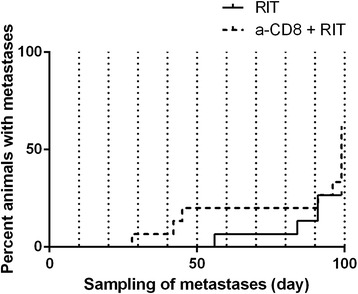
**Curve of metastatic disease.** Fraction of rats with observed metastatic disease in RIT (*solid line*) and anti-CD8 + RIT group (*dashed line*) during the study period including results of autopsy at the end of the study. Day 0 = day for injection of ^177^Lu-DOTA-BR96 treatment.

Five of the 15 animals in the anti-CD8 + RIT group were sacrificed due to loss of body weight (on days 28, 42, 45, 91, and 96), and metastases were verified in all these animals at subsequent autopsy. The autopsy performed at the end of the study revealed metastases in 4 additional animals, but the remaining 6 animals were free from detectable metastases. Four of the 15 animals in the RIT group were sacrificed due to loss of body weight (on days 56, 84, 91, and 91), metastases being verified at autopsy. All the remaining animals in this group were free from detectable metastases at the end of the study (there were no significant difference between the two groups, Mantel-Cox test *p* = 0.11); in the RIT group, it is expected to find metastases up to 100 days after administration of radioimmunoconjugate according to our previous study [21], indicating that metastases do not develop later. Thus, 99 days after injection of the radioimmunoconjugate, 11 animals in the RIT group were free from detectable metastases, while only 6 were free from detectable metastases in the anti-CD8 + RIT group (Figure 2, Table 1).

**Table 1:** **Metastases in animals after treatment**

**Group**	**Metastatic disease**	**Metastatic findings at autopsy, day 99**	**Free from detectable metastases**
Anti-CD8 + RIT, *n* = 15	5	4	6
RIT, *n* = 15	4	0	11

Number of animals with symptomatic metastatic disease (verified at autopsy), metastatic findings at autopsy on day 99 (end of the study), and the animals free from detectable metastases in the two groups (anti-CD8 + RIT and RIT).

The hazard for developing metastases at day 99 was 2.5 times higher in the anti-CD8 + RIT group than in the RIT group [hazard = 2.5, 95% confidence interval (CI) 0.76 to 8.0, *p* = 0.13]. The corresponding odds ratio was 4.1 [[(9/6)/(4/11)] 95% CI 0.88 to 19, *p* = 0.07]. Metastases were found in surgical scar (1 animal), abdominal lymph nodes (2 animals), mesentery (2 animals), retroperitoneal space (2 animals), abdominal cavity (6 animals), lungs (7 animals), and upper thoracic lymph nodes (11 animals). No difference in location was observed between the two treatment groups. No animal was sacrificed due to the side effects of treatment.

### Myelosuppression and the effect of anti-CD8

Both 0.5 and 1.0 mg anti-CD8 antibodies completely depleted the CD3^+^CD4^−^CD8^+^ lymphocytes in the blood on the day after injection. Eight days after injection, the CD3^+^CD4^−^CD8^+^ lymphocytes started to recover, and there was still no difference between the two dosages. For 0.5 mg, the average %CD3 + CD8+ of CD3+ lymphocytes 3 days before administration was 11 and for 1 mg 10.3. The corresponding results the day after administration was 0.09 and 0.06, respectively, 11 days after administration 5.6 and 5.9 and 25 days 9.2 and 8.0. We decided therefore for the main study to repeat the administration but with a lower amount of anti-CD8 given on day 9 to prolong the depletion of CD3^+^CD4^−^CD8^+^ lymphocytes.

**Figure 3:**
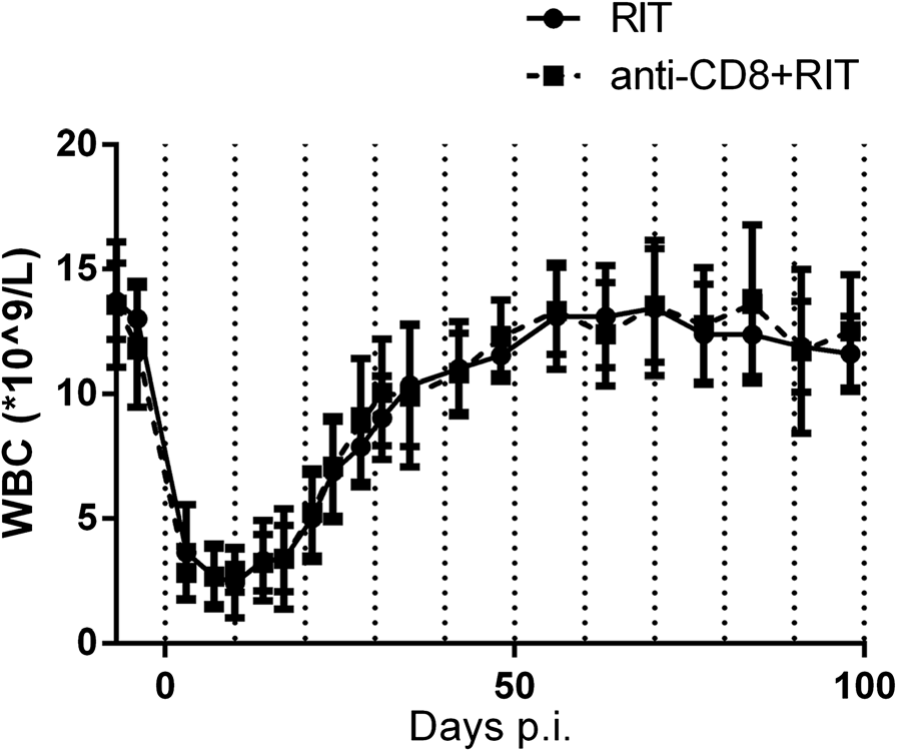
**Myelosuppression after treatment.** White blood cells in animals treated with RIT (*solid line*) and animals treated with anti-CD8 + RIT (*dashed line*). The *error bars* represent the standard deviation.

**Figure 4:**
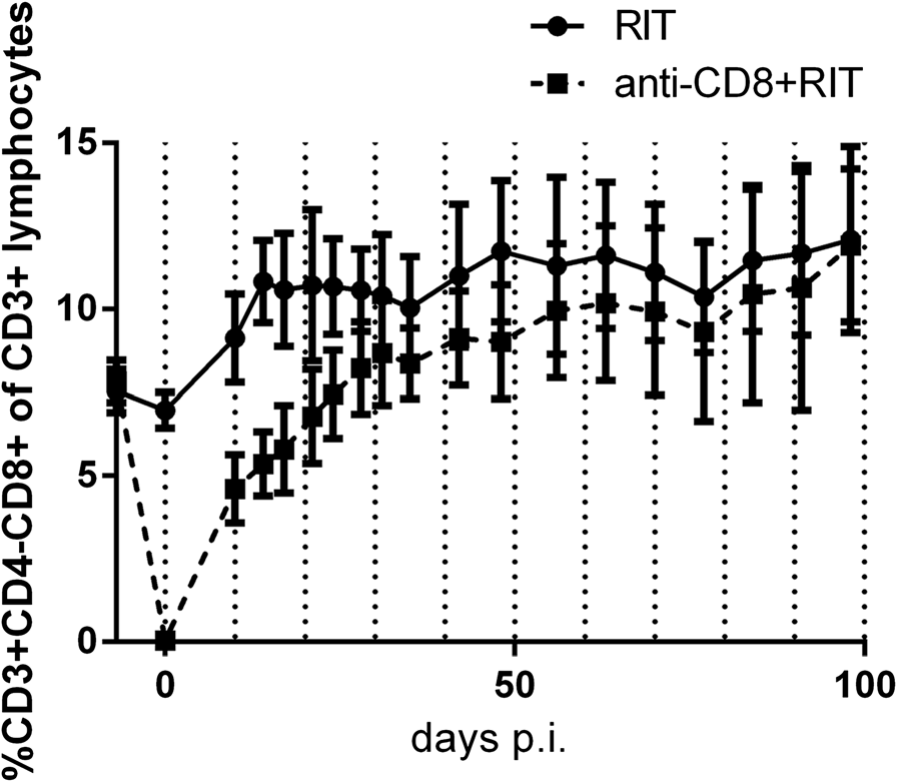
**Effects on CD8** ^**+**^ **cells after treatment.** The average of %CD3^+^CD4^−^CD8^+^ of CD3^+^ lymphocytes in the RIT group (*solid line*) and in the anti-CD8 + RIT group (*dashed line*). The *error bars* represent standard deviation.

In the main study, the myelosuppression was similar in the two groups, both in terms of severity and of duration; see Figure 3. All animals in the anti-CD8 + RIT group exhibited depletion of CD3^+^CD4^−^CD8^+^ lymphocytes on day 0. The RIT group showed an increase in %CD4^−^CD8^+^ of all CD3^+^ lymphocytes as a result of the therapy. At the end of the study, both groups had similar values of %CD4^−^CD8^+^ of CD3^+^ lymphocytes, as can be seen in Figure 4.

**Figure 5:**
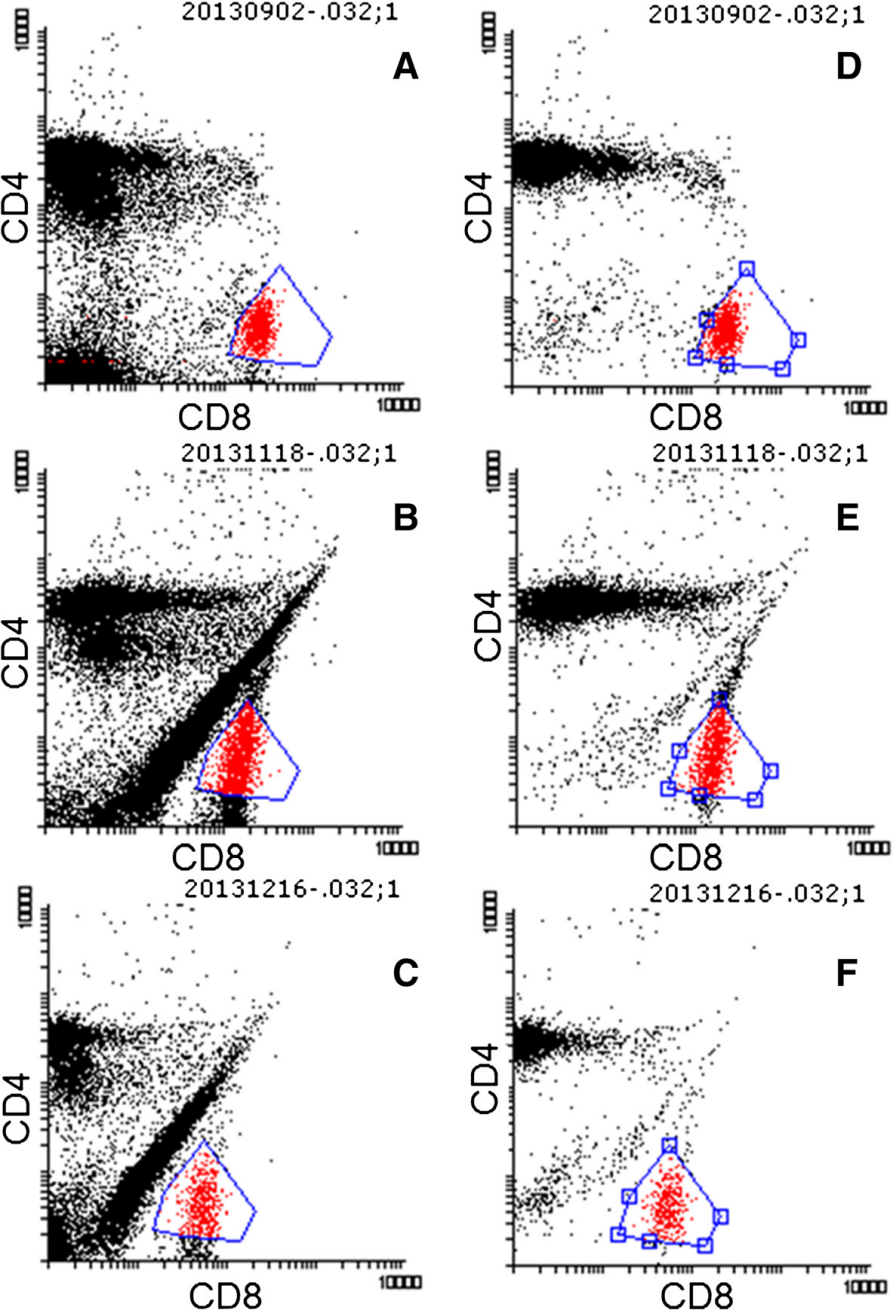
**Flow cytometric findings during the recovery of CD8** ^**+**^ **cells in anti-CD8-treated animals.** Dot plots from the same animal treated with anti-CD8 + RIT, CD4^−^CD8^+^ cells *labeled red*. **A** to **C** illustrates all lymphocytes, and **D to F** illustrates CD3^+^ lymphocytes. **A**, **D** Untreated, 1 week before administration of radioimmunoconjugate (day −7). **B**, **E** 70 days after injection of radioimmunoconjugate. **C**, **F** 98 days after injection of radioimmunoconjugate.

The majority of the animals in the anti-CD8 + RIT group showed a linear relationship between CD4^+^ and CD8^+^ (double-positive lymphocytes) within the lymphocyte gate during the recovery of CD8^+^ cells, as shown in Figure 5. The majority of these cells were not CD3^+^, and this cell type (CD3^−^CD4^+^CD8^+^) was seen from the time of recovery of lymphocytes and the duration varied from a couple of weeks until the end of the study period. This type of cell was not seen in animals receiving RIT only.

## Discussion

We have found that an early depletion of CD8-positive cells during RIT seemed to result in the development of metastases, as demonstrated by the earlier and higher frequency of metastases in the anti-CD8 + RIT group (hazard = 2.5, *p* = 0.13, and odds ratio = 4.1, *p* = 0.07). At the end of the study (99 days post therapy), 6 of 15 animals treated with anti-CD8 + RIT were free from distant metastases, compared to 11 of 15 animals treated with RIT. However, the depletion of CD8-positive cells did not affect the response of established tumors after RIT; probably, the response of the tumor was dependent on the irradiation rather than induction of an immune response. Our interpretation is that CD8-positive cells may play a more important role in preventing or delaying the development of microscopic metastases than in the regression of already established tumors.

Treatment with anti-CD8 + RIT did not increase myelosuppression compared to RIT alone in the present study. We found that anti-CD8 + RIT induced double-positive lymphocytes (CD4^+^CD8^+^) during the recovery of the lymphocytes, seen as a linear relationship between CD4^+^ and CD8^+^ cells within the lymphocyte gate in flow cytometry analyses (Figure 5). This may indicate a leakage of immature T cells from the thymus. CD4^+^CD8^+^ cells are a normal finding in the thymus [22-26]; they can be present in the circulation in healthy individuals [27,28] and at a higher proportion in some diseases, *e.g*., autoimmune conditions [26,28]. It has been reported that these cells can be induced by antibodies to CD8 or CD4 [29], but it is not known whether these CD4^+^CD8^+^ cells are functionally active. We did not observe any symptoms or signs of autoimmune disorders during the 102 days of follow-up after the first injection of anti-CD8. In the RIT group, no double-positive cells (CD4^+^CD8^+^) were detected, but the total number of CD8^+^ cells was also reduced in the RIT group due to myelosuppression by irradiation from the RIT. However, the reduced number was still sufficient to prevent to a certain extent metastases, as the animals in the anti-CD8 + RIT group had a higher risk of developing metastases. The second administered dose of anti-CD8 was given with the intention to prolong the depletion of CD8-positive cells and delay the recovery. The lack of further depletion of the second administration might be due the possibility of the induction of rat anti-mouse antibodies, which were unfortunately not evaluated.

In the present study, we administered an activity of 400 MBq/kg body weight, which results in mean absorbed dose rates of approximately 0.2 to 0.6 Gy/h at the maximal uptake, 24 h p.i. [30]. To the best our knowledge, no study has evaluated the effect of CD8 lymphocytes on the therapeutic outcome of RIT. Studies evaluating the role of CD8-positive cells during external-beam radiation therapy have been performed, but further evaluation of the effects of fractionation and the administered dose must be carried out [31,32]. Gough *et al.* have shown that a single fraction of 20 Gy in 3LL (Lewis lung carcinoma)-bearing mice significantly decreased the number of CD8-positive tumor-infiltrating cells within 2 days after radiation, but with a significantly higher fraction of CD8-positive cells infiltrating the tumor 7 days after radiation [10]. They also found that depletion of CD8 T cells prior to radiotherapy (20 Gy given three times over 10 days) significantly decreased the median survival compared to radiotherapy without CD8 depletion [10]. Lee *et al.* showed that the therapeutic results of a single dose of 20 Gy in B16 tumors were almost abrogated by CD8 depletion. A single fraction of irradiation has been found to significantly increase tumor regression in immunocompetent mice, but not in T cell-deficient mice, and an increase in T cells infiltrating the tumor microenvironment was seen 1 to 2 weeks after the treatment [11]. In another study, TUBO tumors in SCID mice were found to be less responsive to radiation than TUBO tumors in immunocompetent BALB/c mice [9]. The tumors in these immunocompetent animals treated with anti-CD8 antibody and radiation continued to grow, while tumor growth was inhibited in animals treated with radiation only [9]. Yoshimoto *et al.* reported a significant decrease in the therapeutic efficiency of radiation and significantly shortened survival time in animals treated with 30 Gy and anti-CD8 [12]. The findings of these animal studies demonstrate that CD8-positive cells are involved in the therapeutic effects of radiation.

There can be a difference in the biological effects of external and internal radiation which can influence CD8-positive cells differently. External radiation is administrated at high dose rate during a short time, often repeated, whereas RIT radiation is administrated at a low-dose rate continuously for a longer time. This continuous decay may also inhibit the lymphocyte activity within the tumor. In the present study, we focused on CD8-positive cells, but lymphocytes are sensitive to low doses of irradiation [33-35]. Macrophages, NK cells, and DCs are more resistant to radiation [33,34], but their role might also be important in RIT and they should therefore be further investigated.

## Conclusions

The depletion of CD8-positive cells in our syngeneic rat colon carcinoma model seemed to result in a higher frequency of animals developing distant metastases, thus indicating that cytotoxic T cells participate in an immune response to the tumor. This finding indicates that future investigations should be undertaken combining RIT with immunotherapy to delay or prevent development of distant metastases. These cytotoxic T cells did not seem to have any major effect on already established tumors.

## Abbreviations

BCA: bicinchoninic acid
BN: Brown Norway
BSA: bovine serum albumin
DOTA: 1,4,7,10-tetraazacyclododecane tetraacetic acid
DTPA: diethylene triamine pentaacetic acid
HPLC: high-performance liquid chromatography
HSA: human serum albumin
ITLC: instant thin-layer chromatography
PBS: phosphate-buffered saline
PFA: paraformaldehyde
RIT: radioimmunotherapy
